# HUGE pipeline to measure temporal genetic variation in *Drosophila suzukii* populations for genetic biocontrol applications

**DOI:** 10.3389/finsc.2022.981974

**Published:** 2022-09-20

**Authors:** Nathan R. Feltman, Eric C. Burkness, Dominique N. Ebbenga, William D. Hutchison, Michael J. Smanski

**Affiliations:** ^1^ Department of Biochemistry, Molecular Biology, and Biophysics, University of Minnesota, Saint Paul, MN, United States; ^2^ Biotechnology Institute, University of Minnesota, Saint Paul, MN, United States; ^3^ Department of Entomology, University of Minnesota, Saint Paul, MN, United States

**Keywords:** genetic biocontrol, population genetics, amplicon sequencing, CRISPR, SWD

## Abstract

Understanding the fine-scale genome sequence diversity that exists within natural populations is important for developing models of species migration, temporal stability, and range expansion. For invasive species, agricultural pests, and disease vectors, sequence diversity at specific loci in the genome can impact the efficacy of next-generation genetic biocontrol strategies. Here we describe a pipeline for haplotype-resolution genetic variant discovery and quantification from thousands of Spotted Wing Drosophila (*Drosophila suzukii*, SWD) isolated at two field sites in the North-Central United States (Minnesota) across two seasons. We observed highly similar single nucleotide polymorphism (SNP) frequencies at each genomic location at each field site and year. This supports the hypotheses that SWD overwinters in Minnesota, is annually populated by the same source populations or a combination of both theories. Also, the stable genetic structure of SWD populations allows for the rational design of genetic biocontrol technologies for population suppression.

## Introduction

Understanding the population genetics of a pest organism is critical for pest management (1). It can reveal the presence of cryptic species groups that are non-mixing within an otherwise continuous population or identify genetically distinct sub-populations that arise from geographical barriers to gene flow (2–4). Population genetics can inform whether an invading population arose from a single founder population or from repeated introductions, which has important ramifications for long-term management strategies (5). Lastly, an understanding of population genetics can drive important design constraints for genetically engineering biocontrol agents (6–8).

Molecular approaches to population genetics have advanced rapidly from non-sequencing based methods to methods that leverage the array of Next Generation Sequencing (NGS) platforms. NGS methods provide the finest level of resolution, particularly if they are directed towards mitochondrial or nuclear genes that are rich in single nucleotide polymorphisms (SNPs). Amplification of multiple loci from individually-isolated genomic DNA (gDNA) samples provides haplotype-level resolution of population structure (9). In this case, isolating gDNA from each organism separately allows for linkage between haplotypes at different genomic loci to be calculated, but the bottleneck of performing individual gDNA isolations limits the number of organisms that can be practically analyzed. NGS technology enables whole genome SNP comparison from individual or pool-extracted samples of gDNA (10, 11). This can provide information on a massive number of SNPs but the sequencing cost and size of most genomes limits the number of individuals that can be analyzed to hundreds. While this approach is powerful for tracing evolutionary lineages, it is less-well suited for quantifying rare haplotypes.

Deep sequencing of PCR amplicons arising from pooled gDNA samples is an attractive technique for maximizing the sequence coverage at targeted loci in the genome (12). Barcodes added to oligonucleotide primers during NGS library preparation allow multiple samples comprising thousands of individuals to be sequenced in a single Illumina lane (13). PCR enrichment is often used prior to NGS analysis for viral genomes (14). For the identification of rare mutants in plant cultivars, amplicon sequencing of multidimensional pools of gDNA has proven to be a powerful and useful tool. Spiked sequence control experiments have shown that amplicon sequencing can routinely detect SNP variants with frequencies as low as 0.002 as distinguishable from sequencing noise (15). Several computational workflows for mapping reads and quantifying SNP frequencies have been compared head-to-head (12, 16) to understand the bias resulting from data processing methods. Cumulatively, these studies show that amplicon sequencing of pooled gDNAs is an economical and robust method for identifying rare SNPs at target loci.

Here we demonstrate an amplicon sequencing analysis pipeline for Haplotype-resolution analysis of Unique loci by bulk Genomic Extraction (HUGE). We used the HUGE pipeline to perform haplotype-level analysis of >10,000 individual Spotted Wing Drosophila (SWD, *Drosophila suzukii* Matsumura) isolated from two field sites in the North-Central United States across two growing seasons. We targeted SWD as a model pest because of its global impact on fruit production (17, 18), and because it is the focus of genetic biocontrol technology development. We used amplicon NGS and bioinformatic analysis of multiple loci to quantify haplotype frequencies in pools of up to 2,000 flies. The data we generated suggest that genetic biocontrol is feasible for these populations and would face low initial rates of genetic resistance. Our results show no difference in the population genetics either spatially between the two field sites or temporally over two years.

## Materials and methods

### 2018 field collections

Two raspberry farms in Minnesota, US were selected as field sites for SWD trapping. Fifteen Scentry SWD drowning traps were set at each location to collect SWD for 1 or 2 weeks. The traps were baited with Scentry SWD lures (Scentry Biologicals Inc., Billings, MT) and 200 mL drowning fluid to drown and collect SWD. Drowning fluid is 0.5% Palmolive dish soap in tap water. Traps were harvested weekly.

### 2019 field collections

**Figure 1:**
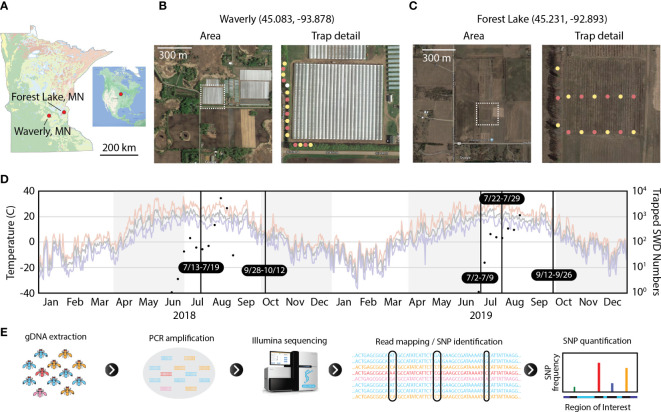
Experimental design for SWD trapping and amplicon sequencing. **(A)** Geographic location of two field sites shown within the boundaries of Minnesota. **(B, C)** Aerial image of raspberry farms and trap locations for Waverly and Forest Lake sites, respectively, for the 2019 collection season. In trap detail images, yellow dots represent apple cider vinegar traps, pink dots represent Scentry traps, and the lone white dot in Waverly is an apple cider vinegar trap monitored by the farmers that was not used in this study. **(D)** SWD population size and daily temperature during the study period. Temperature traces plot daily high (red), mean (grey), and low temperatures (blue). Black vertical lines denote sampling periods for flies used in this study. Black dots show total SWD trapped immediately north of the raspberry plots at Forest Lake. These traps were removed in early September of each year and were not included for sequencing as in **Table 1**. **(E)** Schematic representation of HUGE, the amplicon sequencing and analysis pipeline. Satellite images are from Google Maps.

The same two raspberry farms were used for the 2019 field collections. Fifteen Scentry SWD drowning traps were set at each location to collect SWD for 1 to 3 weeks. Due to high demand for SWD trap lures, Scentry lures were not available for every trap in 2019. For the early July 2019 collections, half of the traps were baited with 200 mL apple cider vinegar (containing 0.5% Palmolive dish soap) without a Scentry SWD lure (**Figures 1B, C**, yellow dots) while the other half were baited with Scentry SWD lures and 200 mL drowning fluid (**Figures 1B, C**, pink dots). For the late July and September 2019 SWD collections, half of the traps were baited with apple cider vinegar with soap and Scentry SWD lures (**Figures 1B, C**, yellow dots) while the other half were baited with Scentry SWD lures and drowning fluid (**Figures 1B, C**, pink dots). Traps were harvested weekly.

### SWD sorting and preservation

Harvested SWD were stored in drowning fluid (water or apple cider vinegar) at 4°C for up to 2 weeks before sorting. SWD were separated out from other fruit fly species. Male SWD were identified by the presence of a single light to dark gray spot on each wing and the presence of two sex combs on either front leg. Female SWD were identified by the presence of the large, serrated ovipositor. Only completely intact adult SWD were kept for sequencing. All loose insect parts, embryos, and larvae were rinsed out of the adult SWD pool and discarded. All SWD from a given location and time of year were combined and preserved in 75% ethanol at -20°C for up to 7 months before gDNA extraction.

### Bulk genomic DNA extraction protocol

SWD from a given location and time of year were divided into pools of up to 250 flies for genomic DNA extraction. Established protocols for homogenizing and extracting gDNA from 50 flies were adapted and scaled up 5-fold (19). All samples for a given location and time of year were combined equimass by fly count such that the proportion of gDNA from each fly was equal in the final gDNA mixture.

### Target gene locations and linkage

Nine target genes were selected based on their expected utility in Engineered Genetic Incompatibility (EGI) (6, 7, 20). Based on the most recent annotated genome assembly of SWD ((21), NCBI Accession #: GCF_013340165.1), three genes are located on the X chromosome, five genes are located on chromosome 2, and one gene is located on chromosome 3. The *eve* promoter is located at 6292.3 to 6292.7 kb on chr 2R. The *hh* promoter is located at 948.1 to 948.5 kb on chr 3R. The *jeb* promoter is located at 11041.0 to 11041.4 kb on chr 2R. The *pyr* promoter is located at 10582.6 to 10583.0 kb on chr 2R. The *upd1* promoter is located at 2077.4 to 2077.8 kb on chr X. The *upd2* promoter is located at 2148.7 to 2149.0 kb on chr X. The *upd3* promoter is located at 2110.7 to 2111.1 kb on chr X. The *wg* promoter is located at 8270.2 to 8270.6 kb on chr 2L. The *wnt4* promoter is located at 8306.6 to 8307.0 kb on chr 2L. Recombination rates across the SWD genome are currently unknown so all recombination rates are estimates based on the corresponding gene locations and recombination rates in *Drosophila melanogaster* (22). Three sets of genes are predicted to be closely linked and are less than one centimorgan (cM) apart: *upd1*, *upd2*, and *upd3*; *jeb* and *pyr*; *wg* and *wnt4*. The *eve* promoter is estimated to be 6.00 to 8.77 cM away from the *jeb* and *pyr* promoters. All other loci are expected to segregate independently as they are estimated to be >50 cM apart or are located on different chromosomes.

### Primer design and PCR

The amplicon primer set included 18 primers designed to PCR amplify the promoters of the *eve*, *hh*, *jeb*, *pyr*, *upd1*, *upd2*, *upd3*, *wg*, and *wnt4* genes in *D. suzukii*. Each primer is between 18 and 26nt long and designed with a Tm of 66.5°C in Q5 polymerase buffer. Amplification was performed using Q5 polymerase (New England Biolabs, NEB). Annealing temperatures were estimated using NEB’s TM calculator (https://tmcalculator.neb.com). The 3' end of the reverse primer for each promoter was designed to bind at least 30 bp downstream of the respective transcription start site (TSS). The SWD TSS as annotated on NCBI was used when this information was available. If the SWD TSS was not annotated on NCBI, the homologous sequence of the *D. melanogaster* TSS was used instead. The *D. melanogaster* promoter region was aligned to the homologous promoter in *D. suzukii* and conserved regions near the *D. melanogaster* TSS were assumed to be the *D. suzukii* TSS. We performed a preliminary NGS sequencing of 500-600 bp amplicon to identify low-variance primer-binding regions to which we designed final forward and reverse primers that would yield amplicons between 400 and 425 bp for each target region (**Supplementary Note 1**). All PCRs were performed using Q5 polymerase. The PCR program used for all nine primer pairs included an initial denature step at 98°C for 30s followed by 35 cycles of 98°C for 10s, 66.5°C for 20s, 72°C for 25s. A final extension step was performed for 120s at 72°C. PCR products were electrophoresed in a 1% agarose gel and extracted using a Zymoclean Gel DNA Recovery Kit. Control PCRs were performed targeting the *wg* promoter using a tenth set of primers, pWg_Control_F and pWg_Control_R. These primers were designed to bind to highly conserved regions between SWD and *D. melanogaster* to generate a 387 bp amplicon that included the annotated TSS of both SWD and *D. melanogaster*.

### Sample preparation for Illumina sequencing

Nanodrop was used to quantify the concentration of PCR product from each gel extraction. A total of 110 amplicon pools were created; two sampling locations times five sampling periods and one control gDNA pool, with each gDNA pool being amplified by ten primer pairs. Amplicon pools were combined equimolar into pairs, resulting in 55 samples. Each sample met the sample guidelines given by Azenta and were sequenced using their Amplicon-EZ pipeline.

### DNA library preparation and Illumina sequencing

DNA library preparations, sequencing reactions, and adapter sequences trimming were conducted at Azenta, Inc. (South Plainfield, NJ, USA). DNA Library Preparation were performed using NEBNext Ultra DNA Library Prep kit following the manufacturer’s recommendations (Illumina, San Diego, CA, USA). Briefly, end repaired adapters were ligated after adenylation of the 3' ends followed by enrichment by limited cycle PCR. DNA libraries were validated and quantified before loading. The pooled DNA libraries were loaded on the Illumina instrument according to manufacturer’s instructions. The samples were sequenced using a 2 x 250 paired-end (PE) configuration. Image analysis and base calling were conducted by the Illumina Control Software on the Illumina instrument.

### HUGE data analysis pipeline

**Figure 2:**
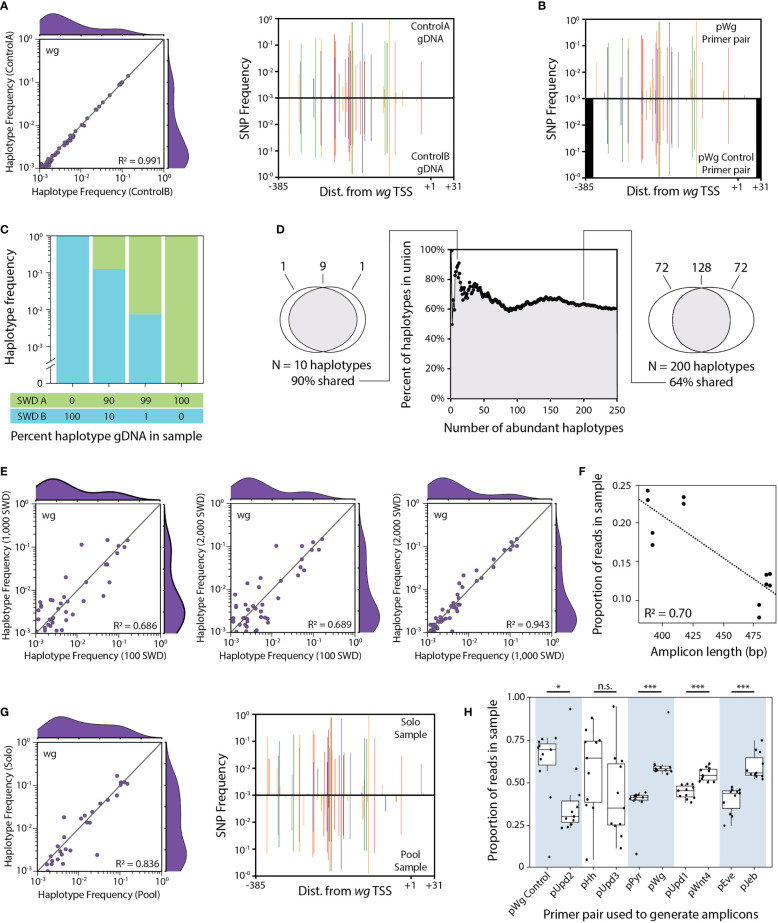
Control experiments performed while developing the HUGE pipeline. **(A)** HUGE performed twice on the same PCR sample. A single PCR was performed using the pWg primer pair and the Waverly July 2019 (l) gDNA pool. This PCR was split into two samples for NGS (**Supplementary Note 2**, samples ControlA and ControlB). Each sample was independently analysed using the HUGE pipeline. Haplotype frequency and mirror SNP plots are used to compare the pair of samples. **(B)** HUGE performed on the same gDNA sample with two different sets of primers. A Waverly July 2019 (l) gDNA pool extracted from 100 flies was used for two independent PCRs. The pWg primer pair was used in the top SNP plot while the pWg_Control primer pair was used in the bottom SNP plot. Black bars indicate promoter regions that were not sequenced by the pWg_Control primer pair. **(C)** HUGE performed on gDNA samples of known haplotype abundance. Four gDNA pools were prepared with different ratios of gDNA from two different inbred SWD lines, SWD A and SWD B. These two lines differ in the *wg* promoter sequence by five discriminatory SNPs. PCR was performed on each gDNA pool using the pWg primer pair, generating samples that were submitted for NGS (**Supplementary Note 2**, samples SpikeIn1 through SpikeIn4). HUGE was performed to determine the relative abundance of the two expected haplotypes. Haplotypes that did not align perfectly to either of the two expected haplotypes were removed. **(D)** Proportion of abundant haplotypes shared between two pools of flies collected from a single time point. Two subsets of flies (containing 100 and 2,000 flies respectively) were made using the pool of flies captured from Waverly July 2019 (l). gDNA was extracted from each subset of flies. PCR was performed on each gDNA pool using the pWg primer pair, generating samples that were submitted for NGS (**Supplementary Note 2**, samples 100Flies and 2000Flies). HUGE was performed on both samples. Each haplotype was rank ordered by relative abundance for each sample. The top N haplotypes were then compared between the two samples for all values of N from 1 to 250. The percent of haplotypes that were the same between the two samples was plotted for each N. **(E)** Comparing haplotype abundance from pooling low, medium, or high numbers of flies sampled from the same location and time point. Three subsets of flies were made using the pool of flies captured from Waverly July 2019 (l). gDNA was extracted from each subset of flies. PCR was performed on each gDNA pool using the pWg primer pair, generating samples that were submitted for NGS (**Supplementary Note 2**, samples 100Flies, 1000Flies and 2000Flies). HUGE was performed on all three samples. All three possible pairwise haplotype frequency plots were made comparing the 100, 1,000 and 2,000 fly samples with each other .**(F)** Amplicon length as a predictor for fraction of reads in a combined sample. Six primer pairs were designed in preliminary testing to amplify six different loci. Expected amplicon sizes ranged from 376 to 496 bp long. PCR was performed on two different gDNA pools (Waverly October 2018 and Forest Lake October 2018). PCR products made from the same gDNA pool were combined equimolar to form two samples that were submitted for NGS. The proportion of reads in each of the samples was plotted for each locus by amplicon length. **(G)** HUGE performed on the same gDNA sample sequenced solo or in a pool of amplicons. Two PCRs were performed using the pWg primer pair and the Waverly July 2019 (l) gDNA pool. One PCR was submitted directly for NGS (**Supplementary Note 2**, sample 2000Flies). The other PCR was combined with another pool of amplicons and submitted for NGS (**Supplementary Note 2**, sample Samp23). Each sample was independently analysed using the HUGE pipeline. Haplotype frequency and mirror SNP plots are used to compare the pair of samples. **(H)** Proportion of reads mapped to either of the two primer pairs pooled per sample. Each of the 55 experimental samples contained amplicons generated from two primer pairs. Each primer pair was always combined with the same partner primer pair (light blue and white shading). The proportion of reads mapping to either of the two partner primer pairs is plotted by read pair. Box and whisker plots are divided by quartiles. For **(A, E, G)**, R^2^ values indicate the coefficient of determination for a linear regression with fixed intercept at (0,0). The linear regression in **(F)** does not have a fixed intercept. For **(H)**, Welch Two Sample t-tests were performed between each pair of pooled gene targets. Statistical significance: *** = p<0.001, * = p<0.05, n.s, not significant. The reference genome to which SNPs were called was published by Chiu et al. 2013 and is available on NCBI (Accession #:GCA_000472105.1) (51).

**Figure 3:**
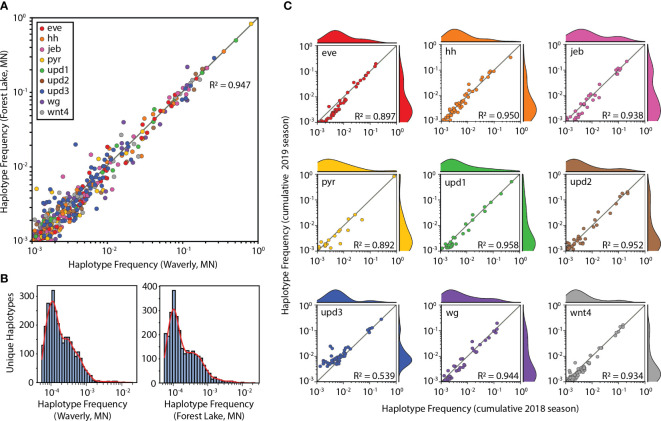
Haplotype frequency variation across space and time. **(A)** Haplotype frequency averaged for 2018 and 2019 growing seasons plotted for Waverly, MN and Forest Lake, MN field sites. Only haplotypes present in both field sites are shown. Data points are colored according to target gene according to the figure legend inset upper left. **(B)** Haplotypes detected in only one of the two field sites, plotted by their population frequency in the Waverly, MN samples (left) or the Forest Lake, MN samples (right). **(C)** Change in population haplotype frequency from 2018 (x-axis) to 2019 (y-axis) growing seasons for each target gene. Target genes are labeled in upper left of each plot and colored according to subplot **(A)**. Histograms above and right of scatter plots show probability distribution function of data points along each axis. R^2^ values in all plots are coefficients of determination calculated with respect to the x = y line. i.e. an R^2^ of 1 would indicate that all haplotypes are present at the same frequency under the conditions of the x-axis as they are under the conditions of the y-axis.

**Figure 4:**
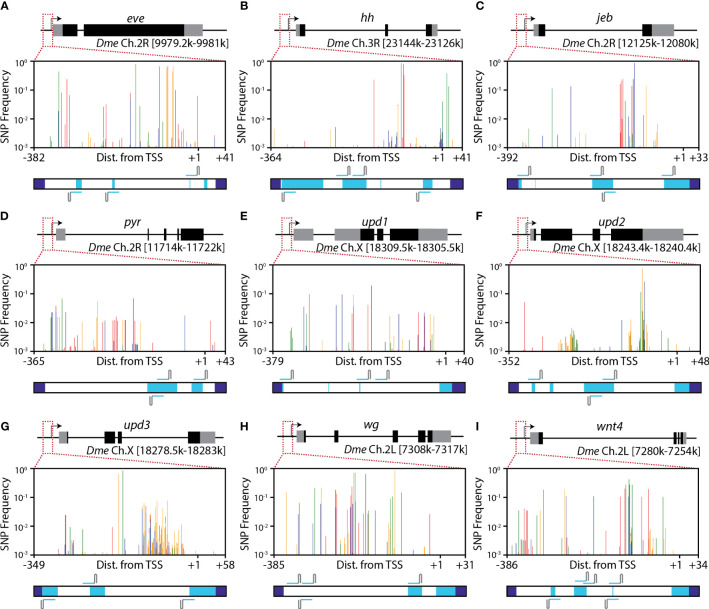
Conserved regions in target promoters. **(A–I)** Mapped SNPs in each of nine putative target genes for developing Engineered Genetic Incompatibility in SWD. Plots depict the frequency of SNPs in a combination of all 10,802 sequenced flies obtained across the 10 collections. SNP frequencies were weighted by the number of flies sequenced from each sample (see **Table 1**) and summed. Vertical lines are colored by the basepair of the SNP with red, orange, green and blue lines depicting **(A, C, G)**, and T SNPs respectively. Below each SNP plot, a horizontal bar spans the entire amplicon. Primer regions (dark blue) depict where primers bound to create the amplicons using PCR. Conserved regions (light blue) indicate that a given base and 15-bp upstream and downstream lack any SNPs at a frequency of 0.002 or greater. For each promoter, at least 3 highly-conserved guide RNAs can be designed for use in Engineered Genetic Incompatibility. All depicted gRNAs lack any SNPs at a frequency of 0.0023 or greater, including any SNPs present in the PAM sequences, and would target at least 98.7% of wild haplotypes.

Compressed fastq files containing raw paired-end reads were received from Azenta. Data analysis utilized computing power from the University of Minnesota Supercomputing Institute. All modules were run using default parameters except where noted otherwise. The raw paired-end reads were processed using *Trimmomatic v0.33* (23) with the parameter MINLEN: 240 to remove short reads. Three modules of *bbmap v38.34* were used: *bbsplit*, *reformat* and *bbmerge* (24). *bbsplit* was used to isolate reads from only one of the two gene targets contained in each sample. *reformat* was used to separate the interleaved output fastq file from *bbsplit* into two fastq files of paired-end reads. *bbmerge* was used to combine each pair of reads into a single haplotype spanning the entire sequenced amplicon. *bbmerge* was run with the parameter pfilter=1, which requires perfect sequence overlap to merge the paired-end reads. *bowtie2 v2.2.4_gcc-4.9.2* (25) was used to align the haplotypes to the reference amplicon sequence and output a sam file. *bowtie2-build* was used to generate the index. The *bowtie2* alignment was performed with the following parameters: -t -f -p 8 –local -L 32 –ma 2 –np 0 –all –reorder. *SAMtools v1.9* (26) was used to convert the sam file to a sorted bam file. *Pilon v1.23* (27) was used to count the frequency of each nucleotide at each basepair in the amplicon from the sorted bam file and output the result as a variant call file (vcf) file. *Pilon* was run with the following parameters: –vcf –flank 0 –fix snps. *usearch v11.0.667* (28) was used to process the haplotype files for each amplicon pool. *usearch -makeudb_usearch* was used to create database files of each reference sequence. *usearch -orient* was used to orient all haplotype outputs in the same direction according to the reference database. *usearch -fastx_uniques_persample* was used to count how many times each unique haplotype appeared in the amplicon pool and output a fasta file. *usearch -fastx_uniques_persample* was run with the following parameters: -sizeout -minuniquesize 5. The fasta file from *usearch* was processed using a *Python v3.8* (29) script (data_extract.py) to extract the haplotype depth data for each haplotype into a separate csv file. A csv metafile for all samples was generated using another python script (hap_extract.py). An *R v4.1.0* (30) script (removing truncated haplotypes.R) was used to remove haplotypes that were formed by using truncated primers during PCR and combine the haplotypes from all samples into a single csv file. A *Python* script (haplotype_analysis_with_rsquared py) was used to generate the haplotype frequency plots in **Figures 2**, **3** using the list of unique haplotypes and their frequencies. A *Python* script (pilonSNPsFIX_HUGE.py) was used to generate the SNP frequency plots in **Figures 2**, **4** using the vcf files output by pilon.

## Results

### Field study design and fly collections

We identified two raspberry farms within 70 km of St. Paul, MN and collected flies during the 2018 and 2019 growing seasons (**Figure 1**). The farm in Waverly, MN is a commercial fruit farm that uses wind tunnels as a primary SWD mitigant. The farm in Forest Lake, MN is a customer-pick raspberry farm. Both farms used regular application of pesticides throughout both growing seasons. SWD collection dates and numbers for each farm are reported in **Table 1**.

**Table 1:** Collection of SWD. SWD caught and sequenced per location and date.

Date	Site	Number of flies
		**Collected (Sequenced)**
July 2018	Waverly	382 (382)
	Forest Lake	808 (808)
	Waverly	119 (119)
October 2018	Forest Lake	100 (100)
July 2019 (e)	Waverly	2069 (1928)
	Forest Lake	1112 (1052)
	Waverly	4490 (2000)
July 2019 (l)	Forest Lake	1881 (1881)
September 2019	Waverly	1288 (1288)
	Forest Lake	1244 (1244)

A total of 13,493 SWD were caught (6,515 male, 6,978 female). Only 10,802 flies were sequenced for the final analysis. Excluded SWD were used for control experiments during protocol development. A total of 13,493 SWD were caught (6,515 male, 6,978 female). Only 10,802 flies were sequenced for the final analysis. Excluded SWD were used for control experiments during protocol development.

### Development of HUGE pipeline and control experiments

For this study, we developed a SNP analysis pipeline for Haplotype-resolution analysis of Unique loci by bulk Genomic Extraction (HUGE) (**Figure 1E**). Genomic DNA was bulk-extracted from flies collected at one time/location. Next, primers specific to the genomic loci of interest were used to generate PCR amplicons from the population gDNA sample. These amplicons were submitted to Azenta for sequencing using their Amplicon-EZ service to generate 2 x 250 bp paired-end reads. PCR products were constrained to 400-425 bp amplicons to ensure sufficient sequence overlap between the paired reads to generate full-length haplotype-level sequence data for the regions of interest.

Prior to quantifying haplotype frequencies between sampled populations, we performed a number of control experiments to minimize the bias in the method. Results from these control experiments are shown in **Figure 2**. We confirmed that error introduced during the NGS sequencing step is minimal by comparing results from the same amplicon sample submitted for sequencing as two independent samples (**Figure 2A**). Primer selection can influence the ability to detect some SNPs (**Figure 2B**), so we amplified all samples with the same primer pair for a given locus.

The relative ratio of haplotypes quantified by HUGE correlates with the molar ratio of template in the gDNA pool (**Figure 2C**). The most abundant haplotypes were similar, but not identical, whether 100 or 2,000 flies collected from a single time point were pooled together (**Figure 2D**). As expected, pooling a larger number of control flies gave haplotype abundances that were increasingly similar to the experimental flies collected from the same location and time (**Figure 2E**).

In preliminary experiments, shorter PCR amplicons (~375 bp) were sequenced more efficiently than long amplicons (~475 bp) (**Figure 2F**). To balance the read depth per locus, we designed all primer pairs to give products within a narrow range of 400-425 bases and only combined two of the ten target genes in a sequencing lane, with an average difference in length of 5 bases. The observed haplotype and SNP frequencies are similar whether a sample was sequenced in isolation versus in a pool with another set of amplicons (**Figure 2G**). There is a read-depth bias that is gene sequence dependent (**Figure 2H**). Each pair of loci were always sequenced together and the ratio of reads for each locus was mostly consistent across samples.

The cumulative outcome of these control experiments lead to a refinement of the HUGE workflow and a final analysis of all 90 experimental amplicon pools (9 genomic loci x 5 sampling periods x 2 field sites) in a single NGS submission. A description of the experimental and control samples that were sequenced in this study is given in **Supplementary Note 2**.

### Sequencing results

Seventy one NGS samples were processed using the HUGE pipeline in this study (**Supplementary Note 2**). A total of 5.8 Gbases of data passed read length and base quality control steps. The average number of read pairs per sample was 89,925. After merging paired reads into haplotypes, an average of 45,006 haplotypes remained per sample (50.0%). The percent of reads merged into haplotypes varied by primer pair (**Supplementary Note 3**). Nine out of ten primer pairs successfully merged 40 to 65% of read pairs into haplotypes. Read pairs generated from amplification of the *upd3* promoter averaged only 4% successful merging into haplotypes. For each gene, the number of read pairs per sample was an excellent predictor for the number of haplotypes per sample (**Supplementary Note 4**).

### Haplotype frequency comparison between field sites and sampling times

Here we tested two field sites located 80 km apart in central Minnesota, United States. We saw a high similarity in the frequency of haplotypes detected at both locations for the nine target loci (**Figure 3A**). Haplotypes that were found at one field site but not the other were predominantly rare haplotypes present at a frequency ≤0.001 (**Figure 3B**). We also saw a high similarity in the frequency of haplotypes detected in both the 2018 and 2019 collection seasons for 8 out of 9 loci (**Figure 3C**). The one locus that was not highly conserved, *upd3*, was prone to artifacts during read merging that obfuscates interpretation of these data (**Supplementary Note 3**). The variance between collection years or between locations was small, with coefficients of determination (*i.e.*, R-squared values) with respect to the x=y line generally greater than or equal to 0.9. When identical samples are processed with the HUGE pipeline, we obtain coefficients of determination of 0.99 (**Figure 2A**). Therefore, we conclude that the wild SWD populations remain highly similar, but not identical, across space and time.

### Sequence variance at target promoter loci

The genomic loci selected for analysis are those in the promoter regions of developmental morphogen genes. These sites are candidate target sites for a population suppression or replacement method based on Engineered Genetic Incompatibility (EGI) (6, 7, 20). The EGI method requires conserved binding sites for a sequence-specific programmable transcription activator (PTA) upstream of a gene that is lethal when over- or ectopically-expressed.

The nine promoter regions we analyzed differed in their level of sequence conservation (**Figure 4**). The promoters of unpaired 2 (*upd2*), unpaired 1 (*upd1*), and pyramus (*pyr*), and had the least sequence variation, as measured by average Shannon Entropy per base pair (**Supplementary Note 5**). The promoters of wingless (*wg*) and Wnt Family Member 4 (*wnt4*) had the highest levels of sequence variation.

Consistent with what we saw previously in published SNP databases for rice and *D. melanogaster* (6), we find highly conserved regions sufficiently large to allow PTA-targeting (>30 bases) in each promoter region we analyzed (**Figure 4**, light blue regions). Specific binding sites for dCas9-based activators derived from *S. pyogenes* require an ‘NGG’ protospacer adjacent motif (PAM) for binding. Out of a total of 324 putative PTA binding sites that contain an ‘NGG’ PAM site, 208 have a summed SNP percentage below 5%, 113 have a summed SNP percentage below 1%, and 33 have a summed SNP percentage below 0.5% (**Supplementary Note 6**).

## Discussion

In this study, we analyzed over 10,000 wild-caught SWD from two field sites in central Minnesota over the course of two raspberry growing seasons. Using the HUGE pipeline, we assessed genetic variation in samples of thousands of SWD across nine genomic loci. Our results show that while the sampled populations contain substantial genetic diversity on the individual level, the genetic structure of these populations remains similar. We discovered highly-conserved regions wherein sgRNAs can bind in more than 99% of the population, facilitating the design of genetic biocontrol approaches such as Engineered Genetic Incompatibility.

The HUGE pipeline is not the first pipeline to utilize next generation sequencing to assess sequence variation of a population. Preparing sequencing libraries using DNA from many independent samples at once has been generally termed Pool-seq (31). Previous studies have developed and utilized amplicon Pool-seq pipelines to discover rare SNPs and Cas9-induced gene edits (12, 16, 32, 33). Most population genetics studies in SWD have also utilized amplicon sequencing approaches (34–36).

However HUGE is the first Pool-seq pipeline to generate both haplotype-resolution data and detect variants present at a frequency as low as 0.001. We achieve this through overlapping paired-end next generation sequencing of up to thousands of individuals at a read depth ranging from 10 to 1,000 per fly. Other pipelines are limited to performing whole-sample variant calling without being able to determine any variant linkage (16, 33). Other pipelines are also limited to sequencing tens or hundreds of individuals per sample (10, 12, 32–36), while we demonstrate that HUGE can be used on thousands of individuals per sample.

Due to its reliance on high-throughput amplicon sequencing, the HUGE pipeline has limitations when linking SNPs or haplotypes between different loci. HUGE provides high sequence resolution at the population level across time and space, but has limitations at the individual level. Amplicons need to be fairly short (less than 425 bp) to ensure there is enough overlap of the paired end reads to generate a continuous sequence for the entire amplicon. HUGE cannot link genetic variation between loci for individual flies. This limitation is shared by previous SWD population genetics studies that also utilized PCR amplification of specific loci (34–36). One SWD population genetics study utilized pooled whole genome sequencing, a method that can provide information about linkage disequilibrium in the population (10). However, information about individual linkage is still limited to the length of a single sequencing read. Only one SWD population genetics study has utilized single fly whole genome sequencing, but sequencing was limited to an average of 6 flies per sampling location (11). The cost of performing such a study may also be prohibitive compared to the cost of performing a HUGE analysis (**Supplementary Note 7**).

As with any NGS project, sequencing errors are present in our data set. When a sequencing error occurs, it is more likely to falsely declare a SNP than to correct a SNP into the reference sequence; the average SNP frequency per basepair is well below 0.75 for all gene promoters (**Supplementary Note 5**), so most sequencing errors must have produced a novel SNP by incorrectly calling one of the three non-reference bases at a given basepair. We required each unique haplotype sequence to be present at least five times to reduce the ability of sequencing errors to create novel haplotypes. Due to the high read depth, we expect that the HUGE pipeline still results in an over-estimation of the number of unique haplotypes and SNPs present in the sampled populations. Despite this, we observe high similarity in haplotype frequency between identical samples (**Figure 2A**), suggesting that our analysis is highly robust to NGS errors.

The ability for HUGE to detect rare haplotypes depends upon the read depth, the number of individuals, and the number of loci sequenced in a sample. During data processing, we remove haplotypes supported by less than five merged read pairs. To identify a haplotype with a frequency of 0.002 from 250 flies (assuming that each haplotype is evenly amplified) we would need at least ten read pairs per individual. Assuming 40% of read pairs successfully merge into haplotypes (the minimum merging percentage which excludes upd3, **Supplementary Note 3**), a sequencing depth of 25 read pairs per individual is likely to detect the rare haplotype. The Amplicon-EZ service guarantees a minimum of 40,000 read pairs per sample (an average of 90,000 read pairs per sample was observed in this study). Therefore, a conservative estimate is that up to 3,200 individuals might be sequenced at one locus to detect haplotypes as rare as 1 in 500.

The sequencing quality for some samples decreases near the ends of some sequencing reads (**Supplementary Note 8**). For this reason, we required perfect sequence matches to merge the paired end reads together. This means that the final 60-100 bases of both reads in a pair match perfectly to form a complete haplotype. Despite this stringent requirement, 53% of experimental read pairs passed this filtering step and were merged into haplotypes; 8.9 million read pairs merged into 4.75 million haplotypes across all 110 experimental samples. The percentage of read pairs that successfully joined into haplotypes correlated with gene target (**Supplementary Note 3**). *upd3* averaged only 4% of haplotypes merging, which led to a low haplotype sequencing depth for this gene. This low success rate of haplotype merging may be an artifact of high genetic variability in the *upd3* promoter sequence, which has a highly repetitive region in its center that spans 80-100 bp. Samples with virtually no genetic diversity had a 96% success rate when merging reads into haplotypes (**Supplementary Note 2**, samples SpikeIn1 through SpikeIn4). However, genetic variability alone does not explain the variation in read merging success rate between target genes (**Supplementary Note 9**).

The HUGE pipeline utilizes PCR amplification to generate the amplicons sequenced using NGS. Therefore, HUGE is subject to four primary sources of bias inherent to PCR; DNA polymerase errors, PCR stochasticity, PCR bias, and template switching (37).

DNA polymerase errors may result in the incorporation of incorrect basepairs during amplification of the target loci. We used Q5 DNA polymerase to generate the amplicons (New England Biolabs, NEB). To our knowledge, Q5 is the highest fidelity DNA polymerase commercially available, boasting a per basepair error rate below 1 in 1.5 million (38). Polymerase errors are not expected to be an appreciable cause of bias in the HUGE pipeline.

PCR stochasticity is the variation in amplification of templates based on abundance, with common templates being preferred over rare templates. If PCR stochasticity were a major factor in the HUGE pipeline, we would expect abundant sequences to be preferentially amplified and become over-represented after analysis. However, haplotype frequencies output by HUGE match the input template frequencies, with rare haplotypes being observed at similar frequencies to their corresponding template (**Figure 2C**). We expect that PCR stochasticity is not substantially biasing the observed haplotype ratios.

PCR bias is the variation in amplification of templates based on sequence variation. Natural sequence variation in the primer binding regions may cause PCR bias to occur. We demonstrated that primer binding bias can cause minor changes in detected SNP frequencies (**Figure 2B**). To minimize this effect, the primers were designed to bind to highly-conserved sequences. Forward primers were designed in regions conserved between *D. melanogaster* and SWD. Forward primers were additionally constrained to conserved sequences discovered during preliminary testing using alternate primers. All reverse primers were designed to bind downstream of the TSS in regions conserved between *D. melanogaster* and SWD.

Template switching is the joining of two different templates into a novel amplicon during PCR. In the HUGE pipeline, these novel amplicons are interpreted as unique haplotypes, but they may not actually exist in nature. This means that HUGE over-estimates the number of unique haplotypes in the sampled populations, similar to what occurs due to NGS errors. Template switching does not alter SNP frequency measurements as no novel SNPs are being created. If substantial template switching did occur in our analysis, we should be unable to observe consistent haplotype frequencies between locations or seasons (**Figure 3**).

While not the primary objective of this study, the haplotype frequencies we measured could be used to interrogate seasonal migration of SWD. Existing evidence supports that SWD overwinter in their adult winter morph form using snow-covered leaf litter and man-made shelters to survive cold winter temperatures (39–42). The hypothesis that SWD can overwinter in Minnesota is further supported by our two-season comparison, as there is strong correlation of SNPs for each gene target between 2018 and 2019. However, we cannot rule out the possibility that flies from the same founder population seed each season’s invasion in Minnesota. The annual reestablishment of an SWD population in Minnesota may be due to a combination of both factors. This could be determined by applying the HUGE pipeline across a larger geographic area.

The HUGE pipeline can also be useful for performing high-throughput population genetics studies in organisms other than fruit flies. Any organism where a large number of individuals can be harvested (ex. arthropods, annelids, mollusks, microbes) could be processed through the HUGE pipeline. Additionally, HUGE can be used on organisms where genetic samples (somatic tissue, sperm, eggs) can be obtained from many individuals. This applies to plants, amphibians, reptiles, fish, birds and mammals. Therefore, HUGE can be used on all organisms currently being targeted for genetic biocontrol (43).

Genetic biocontrol is a potentially powerful tool to include in integrated pest management plans that combat disease vectors, agricultural pests, and invasive species. Many genetic biocontrol strategies rely on sequence-specific binding to the genome of the target organism. These technologies utilize CRISPR/Cas nucleases or their catalytically inactivated (dCas9 or nCas9) derivatives to direct DNA binding of biocontrol components. Sequence diversity of the sgRNA binding sites in wild populations would likely diminish the efficacy of these Cas9-based technologies. Cas9-directed gene drives rely on conserved sgRNA binding sites to copy-paste the Cas9 nuclease, sgRNA and cargo (44). Toxin-Antidote Recessive Embryo (TARE) and Cleave and Rescue drives rely on sequence-specific Cas9 nuclease activity to inactivate an essential gene while delivering a functional, resistant allele with the cargo (45, 46). Hybrid lethality using Engineered Genetic Incompatibility (EGI) requires sequence-specific binding of a dCas9 activator (7, 20, 47). Although not dependent on Cas9, engineered Medea gene drives rely on sequence specificity to induce lethality *via* miRNA-directed mRNA degradation (48, 49). Homing endonuclease gene drives that utilize non-Cas9 nucleases (ex. TALENs, Zinc-finger nucleases, meganucleases) would still be dependent on sequence specificity. All of these technologies must be designed to target conserved regions to maximize their effectiveness and reduce the rate of evolved resistance. A population genetics study should be performed on any target population prior to the use of these strategies in the field. Ideally, such a study would be performed in any geographical areas that a biocontrol agent would eventually be released for a population suppression or population replacement campaign.

It is unknown if a single SNP is sufficient to prevent lethality caused by the EGI mechanism. Our identification of guide RNA target regions (**Figure 4**) is conservative and assumes that a single SNP can prevent the programmable transcription activator (PTA) used in EGI from causing hybrid lethality. dCas9 binding data suggests that binding can still occur, despite having one or two SNPs in the sgRNA sequence (50). The strength of binding depends on the guide RNA sequence and SNP location within the guide, with SNPs further from the PAM being permissive to stronger dCas9 binding. This may allow the dCas9 activator present in EGI to cause lethality even if one or two SNPs are present in the PTA binding site.

We did not select target loci by prioritizing sequence diversity to generate the most discriminatory data for a population genetics study. Instead, we looked specifically at genomic loci that would be important for the design and construction of SWD biocontrol agents based on the EGI or related sex-sorting incompatible male system (SSIMS) approach (7, 47). Each of the nine genomic loci we sequenced display variable and conserved regions, and there is substantial sequence-level diversity at each locus. Additionally, smaller conserved regions are present in each promoter that facilitate the design of EGI or SSIMS SWD strains that would likely be effective biocontrol agents.

Overall, we collected over 13,000 SWD in the North-Central United States from two locations across five collection periods spanning two years. Using this massive SWD sample, we developed and applied the HUGE pipeline and assessed genetic variation across nine genomic loci. Our results show that the genetic structure of these SWD populations remain similar, both temporally and spatially. The HUGE pipeline also revealed that genetic variation is present between individuals of each population. Despite this genetic diversity, we discovered possible dCas9-sgRNA binding sites that are predicted to be present in over 99% of SWD in these populations. These findings support the continued development of genetic biocontrol based on EGI or SSIMS for suppression of SWD.

## Data availability statement

The datasets presented in this study can be found in online repositories. The names of the repository/repositories and accession number(s) can be found below: https://www.ncbi.nlm.nih.gov/, BioProject number (PRJNA876646).

## Author contributions

MS and WH conceptualized the study. MS and WH acquired funding. NF, EB, and DE established protocols. NF performed experiments. NF and MS analyzed data. NF and MS wrote the manuscript. All authors contributed to the article and approved the submitted version.

## Funding

Funding for this project, including support to NF, was provided by the Minnesota Invasive Terrestrial Plants and Pests Center, through the Environment and Natural Resources Trust Fund, as recommended by the Legislative-Citizen Commission on Minnesota Resources (LCCMR). EB and DE were funded primarily by the Rapid Agricultural Response Fund, Award 0081527, *via* the Minnesota Agricultural Experiment Station, University of Minnesota.

## Acknowledgments

We thank the growers at the Waverly (Untiedt’s Vegetable Farm) and Forest Lake (The Berry Patch), Minnesota, for allowing us to collect SWD samples from their fields. We acknowledge the Minnesota Supercomputing Institute (MSI) at the University of Minnesota for providing resources that contributed to the results reported within this paper. We thank Nathan Myslicki for his assistance with counting and sorting SWD.

## Conflict of interest

The authors declare that the research was conducted in the absence of any commercial or financial relationships that could be construed as a potential conflict of interest.

## Publisher’s note

All claims expressed in this article are solely those of the authors and do not necessarily represent those of their affiliated organizations, or those of the publisher, the editors and the reviewers. Any product that may be evaluated in this article, or claim that may be made by its manufacturer, is not guaranteed or endorsed by the publisher.
