# Supplementary material for: HUGE pipeline to measure temporal genetic variation in Drosophila suzukii populations for genetic biocontrol applications
Source: Front Insect Sci. 2022 Sep 20;2:981974. doi: 10.3389/finsc.2022.981974 (PMC10926429; doi:10.3389/finsc.2022.981974)
Supplement: Supplementary file 1 [file DataSheet_1.pdf]

Supplementary Information for:

HUGE pipeline to measure temporal genetic variation in *Drosophila suzukii* populations for genetic biocontrol applications
Feltman et al.

This PDF file includes:

Supplementary Notes 1 to 9
References

Supplementary Note 1: Primers used in this study

Name	Sequence
pEve_F	GCCATATCATTCTTGATGAAGCCG
pEve_R	GTAGGCAGTTGTTGACTGTGC
pHh_F	AGCAGAAAGGCCTAGGGTTC
pHh_R	TGTTTGCTGTTTCTCTTGATTGGG
pJeb_F	GGATAACCCAGTGAAAAGTGCATT
pJeb_R	CGGTCAGCGGGAAAAAGTC
pPyr_F	GAACCCAAAAATCTGCCCACTTAG
pPyr_R	ATTGGCTTTGGTTATCCGTTGG
pUpd1_F	CGGATCCTGGCCAACTCTAC
pUpd1_R	TTCGAATTTTGGATTTTGAGCTCTGG
pUpd2_F	CGGCTTACAAATCTTCCAGCG
pUpd2_R	TCATGATGACCAGCAGCAACTG
pUpd3_F	TCTCCCTGTCTCCGTCCC
pUpd3_R	GATTCCGATCCGATCGAACG
pWg_F	AGATTGCGCAAATAATCCGGC
pWg_R	ATTCGAGCGGAGGAGTGAAG
pWg_Control_F	GGCAATACAATCGATTACACCG
pWg_Control_R	GGAGTGAAGATTGTTGCCCG
pWnt4_F	GTAGCTGGGATTGGGATTCG
pWnt4_R	GTGCGTCCATCAGCTCGTTC

Supplementary Note 2: List of all Amplicon-EZ samples analyzed using the HUGE pipeline

Supplementary Note 2. The following is a table of all 71 experimental samples and their contents generated in this study. The Sample column lists the name of the sample data. Samp1-55 were the experimental samples that contained amplicons generated from two different primer pairs to more efficiently use the read depth. The Primer Pair column lists the set of primers that were used to generate amplicons for that sample (primer sequences provided in Supplementary Note 1). The Site column lists the location where the sequenced SWD were captured. Rows that read "Lab Stock" were sourced from one or two inbred lines of SWD produced in the lab. The Date column lists the date when the sequenced SWD were captured. Rows that read "Control" were from a subset of SWD captured in July 2019 (l) that were not included in the primary analysis. The Read Pairs column lists the number of read pairs that were of high quality and sufficient length to be used for haplotype generation. The Total Haplotypes column lists the total number of haplotypes that were successfully generated by the HUGE pipeline. Each haplotype is created by merging a set of paired reads with perfect agreement at each base of the overlapping sequence. The success rate of paired read merging by primer pair is plotted in Supplementary Note 3. The Unique Haplotypes column lists how many unique haplotypes were extracted by the HUGE pipeline. The number of total haplotypes and the number of unique haplotypes correlate linearly (Supplementary Note 4).

Sample	Primer Pair	Site	Date	Read Pairs	Total Haplotypes	Unique Haplotypes
SpikeIn1	pWg	Lab Stock	-	132,804	127,499	464
SpikeIn2	pWg	Lab Stock	-	107,902	102,955	390
SpikeIn3	pWg	Lab Stock	-	123,252	119,070	434
SpikeIn4	pWg	Lab Stock	-	103,371	98,905	356
ControlA	pWg	Waverly	July 2019 (l)	125,363	113,264	688
ControlB	pWg	Waverly	July 2019 (l)	180,729	160,089	840
ControlC	pWg	Waverly	Control	390,911	23,979	290
ControlD	pJeb	Waverly	Control	237,847	22,990	212
ControlD	pWg Control	Waverly	Control	259,718	4,344	48
ControlE	pJeb	Waverly	Control	173,306	16,167	162
ControlE	pWg Control	Waverly	Control	190,582	3,048	42
ControlF	pWg Control	Waverly	October 2018	293,264	3,868	66
100Flies	pWg	Waverly	Control	45,337	32,443	291
250Flies	pWg	Waverly	Control	42,244	31,011	236
500Flies	pWg	Waverly	Control	43,429	31,581	251
1000Flies	pWg	Waverly	Control	46,129	33,765	295
2000Flies	pWg	Waverly	July 2019 (l)	45,361	33,255	291
ControlFlies	pWg	Waverly	Control	73,423	53,105	527
Samp1	pUpd2	Waverly	Control	54,845	23,318	233
Samp1	pWg Control	Waverly	Control	39,071	24,627	124
Samp2	pHh	Waverly	Control	60,029	35,174	278
Samp2	pUpd3	Waverly	Control	102,230	4,549	111
Samp3	pPyr	Waverly	Control	81,463	52,797	413
Samp3	pWg	Waverly	Control	107,715	72,845	483
Samp4	pUpd1	Waverly	Control	45,849	26,005	140
Samp4	pWnt4	Waverly	Control	65,029	41,851	394
Samp5	pEve	Waverly	Control	76,124	44,219	316
Samp5	pJeb	Waverly	Control	89,108	50,421	291
Samp6	pUpd2	Waverly	July 2018	79,273	32,998	306
Samp6	pWg Control	Waverly	July 2018	105,411	67,225	317
Samp7	pHh	Waverly	July 2018	71,196	39,765	360
Samp7	pUpd3	Waverly	July 2018	105,481	4,386	119
Samp8	pPyr	Waverly	July 2018	84,387	54,846	455
Samp8	pWg	Waverly	July 2018	122,838	83,504	579
Samp9	pUpd1	Waverly	July 2018	64,680	35,731	216
Samp9	pWnt4	Waverly	July 2018	67,233	40,729	423
Samp10	pEve	Waverly	July 2018	48,540	27,601	257
Samp10	pJeb	Waverly	July 2018	111,334	59,355	382

Sample	Primer Pair	Site	Date	Read Pairs	Total Haplotypes	Unique Haplotypes
Samp11	pUpd2	Waverly	October 2018	62,549	28,226	297
Samp11	pWg Control	Waverly	October 2018	127,016	83,220	496
Samp12	pHh	Waverly	October 2018	57,528	32,254	246
Samp12	pUpd3	Waverly	October 2018	105,032	4,709	116
Samp13	pPyr	Waverly	October 2018	81,565	54,591	414
Samp13	pWg	Waverly	October 2018	110,502	75,930	563
Samp14	pUpd1	Waverly	October 2018	81,804	44,747	254
Samp14	pWnt4	Waverly	October 2018	109,011	67,324	624
Samp15	pEve	Waverly	October 2018	85,400	50,215	375
Samp15	pJeb	Waverly	October 2018	108,731	59,305	346
Samp16	pUpd2	Waverly	July 2019 (e)	43,721	19,676	232
Samp16	pWg Control	Waverly	July 2019 (e)	78,073	51,972	278
Samp17	pHh	Waverly	July 2019 (e)	110,343	57,557	442
Samp17	pUpd3	Waverly	July 2019 (e)	38,918	1,416	50
Samp18	pPyr	Waverly	July 2019 (e)	71,026	43,194	316
Samp18	pWg	Waverly	July 2019 (e)	108,884	71,810	516
Samp19	pUpd1	Waverly	July 2019 (e)	63,138	33,889	205
Samp19	pWnt4	Waverly	July 2019 (e)	70,069	41,735	405
Samp20	pEve	Waverly	July 2019 (e)	71,532	39,160	362
Samp20	pJeb	Waverly	July 2019 (e)	87,759	46,419	307
Samp21	pUpd2	Waverly	July 2019 (l)	22,412	9,732	116
Samp21	pWg Control	Waverly	July 2019 (l)	70,889	46,295	198
Samp22	pHh	Waverly	July 2019 (l)	161,294	87,646	606
Samp22	pUpd3	Waverly	July 2019 (l)	21,026	590	27
Samp23	pPyr	Waverly	July 2019 (l)	80,129	50,570	320
Samp23	pWg	Waverly	July 2019 (l)	112,806	76,401	480
Samp24	pUpd1	Waverly	July 2019 (l)	80,038	43,926	220
Samp24	pWnt4	Waverly	July 2019 (l)	81,809	50,571	394
Samp25	pEve	Waverly	July 2019 (l)	55,513	32,162	211
Samp25	pJeb	Waverly	July 2019 (l)	119,950	65,182	352
Samp26	pUpd2	Waverly	September 2019	37,653	15,165	288
Samp26	pWg Control	Waverly	September 2019	109,182	71,372	597
Samp27	pHh	Waverly	September 2019	111,651	61,080	572
Samp27	pUpd3	Waverly	September 2019	36,785	1,193	50
Samp28	pPyr	Waverly	September 2019	69,074	45,763	384
Samp28	pWg	Waverly	September 2019	108,271	73,721	673
Samp29	pUpd1	Waverly	September 2019	54,503	29,810	242
Samp29	pWnt4	Waverly	September 2019	63,563	38,308	565
Samp30	pEve	Waverly	September 2019	76,810	42,110	531
Samp30	pJeb	Waverly	September 2019	94,493	49,125	526
Samp31	pUpd2	Forest Lake	July 2018	203,380	90,606	682
Samp31	pWg Control	Forest Lake	July 2018	13,728	8,740	44
Samp32	pHh	Forest Lake	July 2018	9,771	5,083	65
Samp32	pUpd3	Forest Lake	July 2018	195,040	9,586	221
Samp33	pPyr	Forest Lake	July 2018	7,992	4,638	52
Samp33	pWg	Forest Lake	July 2018	90,309	60,844	423
Samp34	pUpd1	Forest Lake	July 2018	71,477	39,818	217
Samp34	pWnt4	Forest Lake	July 2018	105,707	66,259	529
Samp35	pEve	Forest Lake	July 2018	71,741	39,760	340
Samp35	pJeb	Forest Lake	July 2018	96,666	50,197	325

Sample	Primer Pair	Site	Date	Read Pairs	Total Haplotypes	Unique Haplotypes
Samp36	pUpd2	Forest Lake	October 2018	41,582	19,365	204
Samp36	pWg Control	Forest Lake	October 2018	95,741	65,492	356
Samp37	pHh	Forest Lake	October 2018	94,594	52,464	362
Samp37	pUpd3	Forest Lake	October 2018	74,829	3,354	88
Samp38	pPyr	Forest Lake	October 2018	75,632	47,794	355
Samp38	pWg	Forest Lake	October 2018	102,764	68,627	484
Samp39	pUpd1	Forest Lake	October 2018	62,371	35,015	187
Samp39	pWnt4	Forest Lake	October 2018	99,296	62,370	528
Samp40	pEve	Forest Lake	October 2018	80,734	46,767	368
Samp40	pJeb	Forest Lake	October 2018	95,081	49,864	306
Samp41	pUpd2	Forest Lake	July 2019 (e)	32,674	13,627	154
Samp41	pWg Control	Forest Lake	July 2019 (e)	106,566	69,477	344
Samp42	pHh	Forest Lake	July 2019 (e)	136,485	75,598	525
Samp42	pUpd3	Forest Lake	July 2019 (e)	31,285	1,244	40
Samp43	pPyr	Forest Lake	July 2019 (e)	90,872	58,921	425
Samp43	pWg	Forest Lake	July 2019 (e)	113,245	76,925	411
Samp44	pUpd1	Forest Lake	July 2019 (e)	56,082	32,258	182
Samp44	pWnt4	Forest Lake	July 2019 (e)	67,272	43,167	375
Samp45	pEve	Forest Lake	July 2019 (e)	61,916	35,120	233
Samp45	pJeb	Forest Lake	July 2019 (e)	98,802	53,780	306
Samp46	pUpd2	Forest Lake	July 2019 (l)	23,936	9,511	119
Samp46	pWg Control	Forest Lake	July 2019 (l)	61,424	39,186	158
Samp47	pHh	Forest Lake	July 2019 (l)	135,223	72,846	679
Samp47	pUpd3	Forest Lake	July 2019 (l)	46,858	1,679	71
Samp48	pPyr	Forest Lake	July 2019 (l)	83,635	54,608	394
Samp48	pWg	Forest Lake	July 2019 (l)	122,962	87,239	636
Samp49	pUpd1	Forest Lake	July 2019 (l)	69,868	36,713	184
Samp49	pWnt4	Forest Lake	July 2019 (l)	71,771	42,099	399
Samp50	pEve	Forest Lake	July 2019 (l)	40,787	21,734	135
Samp50	pJeb	Forest Lake	July 2019 (l)	123,769	59,943	373
Samp51	pUpd2	Forest Lake	September 2019	47,357	18,252	340
Samp51	pWg Control	Forest Lake	September 2019	115,425	70,661	519
Samp52	pHh	Forest Lake	September 2019	95,883	49,186	470
Samp52	pUpd3	Forest Lake	September 2019	52,156	1,803	53
Samp53	pPyr	Forest Lake	September 2019	72,742	43,959	344
Samp53	pWg	Forest Lake	September 2019	96,699	61,561	598
Samp54	pUpd1	Forest Lake	September 2019	63,156	31,594	246
Samp54	pWnt4	Forest Lake	September 2019	86,037	49,318	576
Samp55	pEve	Forest Lake	September 2019	85,695	44,988	486
Samp55	pJeb	Forest Lake	September 2019	94,076	45,826	483

Supplementary Note 3: Percent of read pairs merged into haplotypes by primer pair

Supplementary Note 3. Percent of read pairs merged into haplotypes by primer pair. Each point indicates one of the 110 experimental read pools (Supplementary Note 2, Samp1 through Samp55).

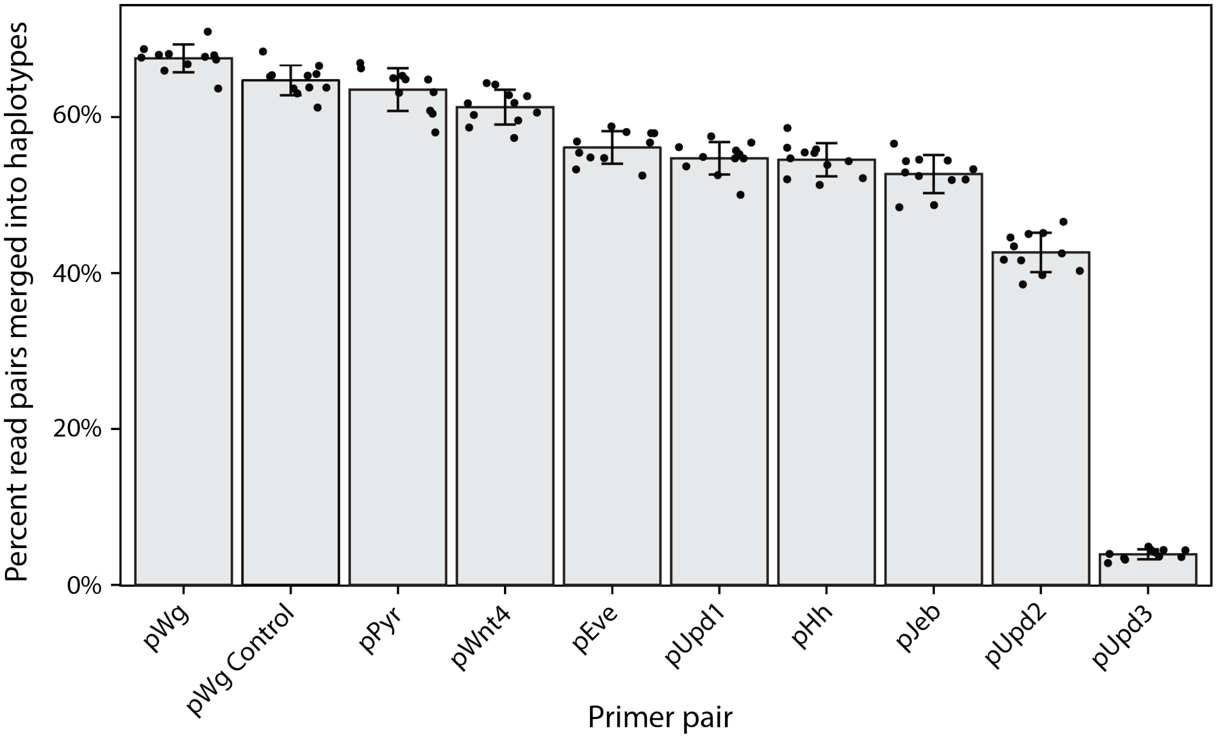

Supplementary Note 4: Linear correlations of total primer pairs, total haplotypes, and unique haplotypes

Supplementary Note 4. Each point indicates one of the 110 experimental amplicon pools (Supplementary Note 2, Samp1 through Samp55). Points are color coded according to primer pair used. R^2 values indicate the coefficient of determination for a standard linear regression model. m indicates the slope of the regression line. (a) For each gene, the total number of read pairs correlates linearly with total haplotypes detected. (b) The number of total haplotypes and the number of unique haplotypes correlate linearly across all genes. wgC; pWg Control.

a

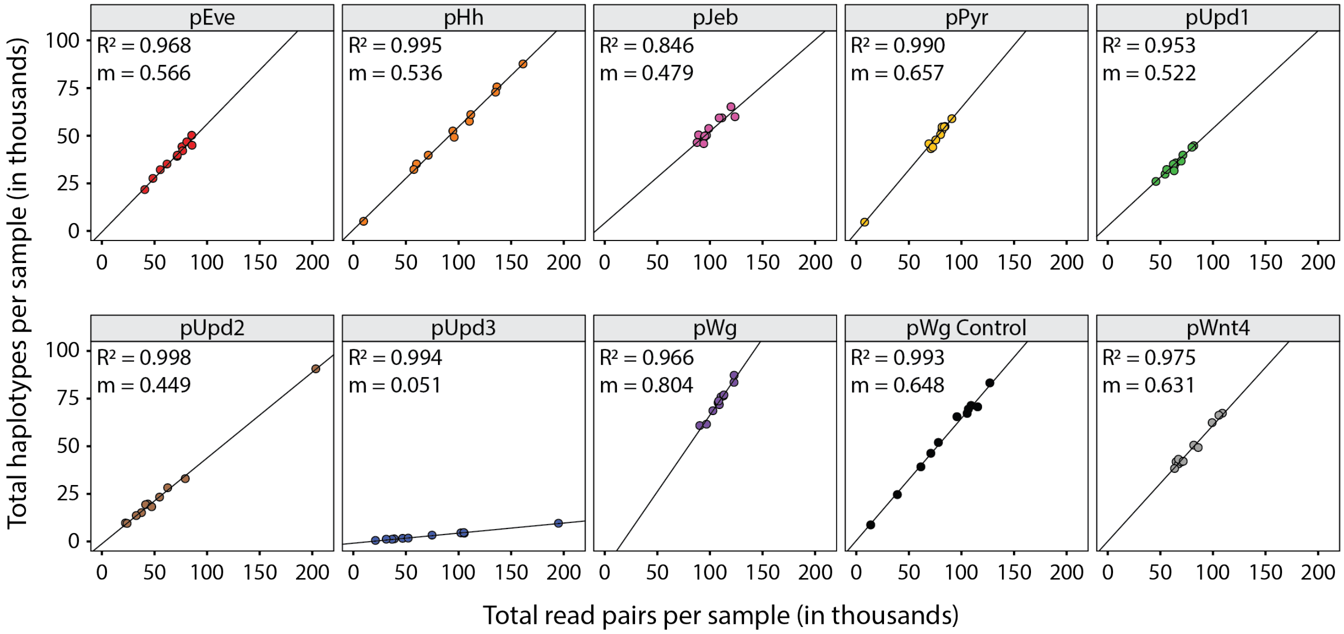

b

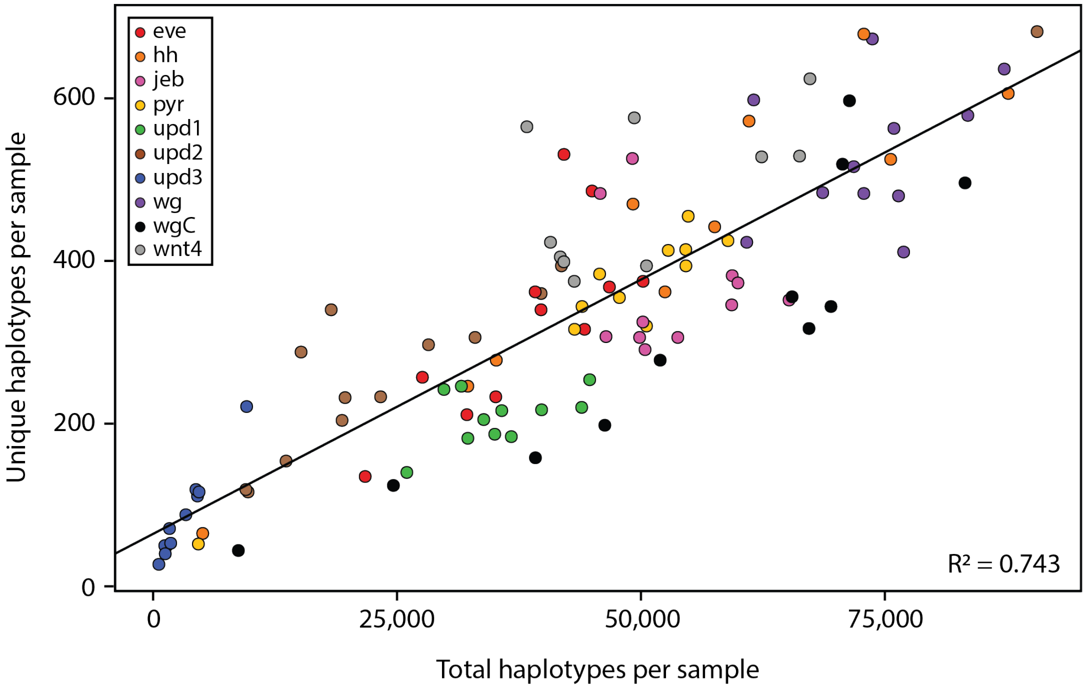

Supplementary Note 5: Quantitative measures of sequence diversity by sequencing locus

Supplementary Note 5. Shannon Entropy was calculated for each base position as $\sum_{N=1}^{3} -P(N)log_2 P(N)$ where $P(N)$ was the SNP frequency of each of the three basecalls that differed from the reference sequence (1). By convention, $log_2(0) = 0$. The reference genome sequence was that of an inbred SWD line established by a single female originally captured in Watsonville, California, United States in September 2009 (NCBI Accession #: GCA_000472105.1) (2). The SNP frequency was calculated for each base position as $\sum_{N=1}^{3} P(N)$. Per bp averages were calculated by summing the respective calculations for a gene promoter and dividing by the expected amplicon length in bp. All calculations use the weighted SNP frequencies from all 10 field collections as described in Figure 4.

Gene promoter	Average Shannon Entropy per bp	Average SNP freq per bp	Percent of bases >0.01 SNP freq
wg	0.0247	0.0131	7.212
wnt4	0.0227	0.0074	4.762
eve	0.0185	0.0127	4.012
jeb	0.0151	0.0060	2.824
upd3	0.0148	0.0057	4.177
hh	0.0121	0.0076	1.975
pyr	0.0113	0.0017	6.373
upd1	0.0112	0.0022	3.819
upd2	0.0103	0.0037	2.000

Supplementary Note 6: Number of targetable sites at various SNP threshold levels

Supplementary Note 6. The summed SNP thresholds used are 0.1%, 0.2%, 0.5%, 1%, 5% and 10%. (a) Percentage of targetable sites below a given summed SNP threshold for each gene target. The x axis is in log2 scale. Points are slightly offset for visibility. The percent of targetable regions increases as the summed SNP threshold increases. (b) The number of PTA targeting sites below a given threshold.

a

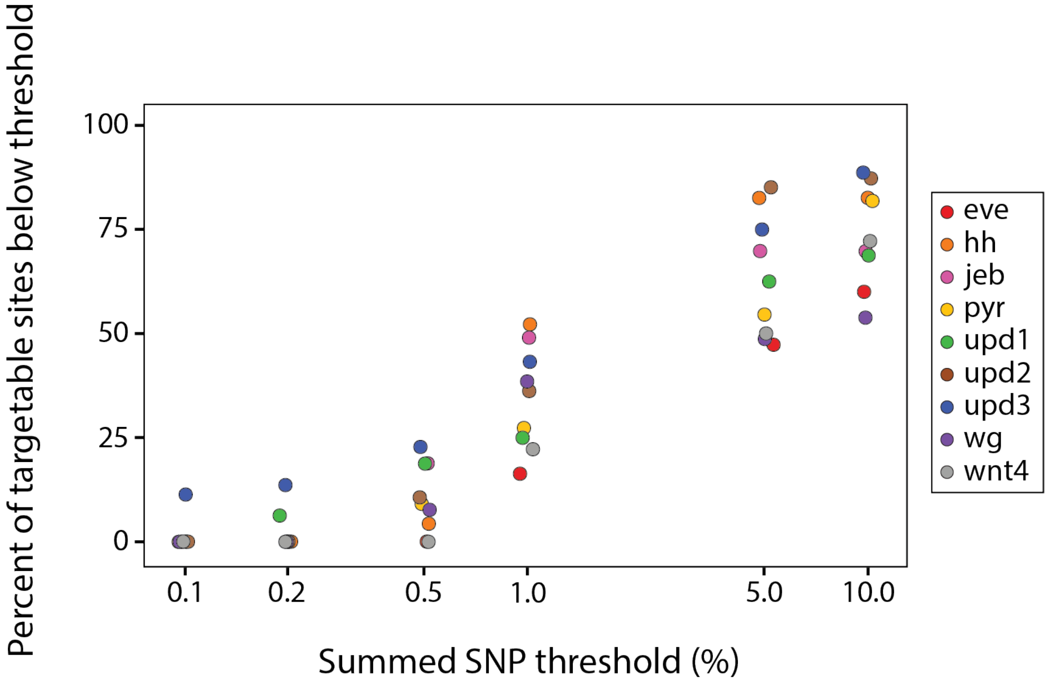

b

Gene promoter	Total guide sites	Guide sites below summed SNP threshold					
		0.10%	0.20%	0.50%	1%	5%	10%
eve	55	0	0	0	9	26	33
hh	23	0	0	1	12	19	19
jeb	53	0	0	10	26	37	37
pyr	11	0	0	1	3	6	9
upd1	16	0	1	3	4	10	11
upd2	47	0	0	5	17	40	41
upd3	44	5	6	10	19	33	39
wg	39	0	0	3	15	19	21
wnt4	36	0	0	0	8	18	26

Supplementary Note 7: Cost breakdown of performing a HUGE analysis

Supplementary Note 7. The cost breakdown of performing a HUGE analysis. The following table lists the costs for comparing sequence diversity at 5 chromosomal loci between 2 populations of 1000 organisms each. Some of these costs will scale linearly with the complexity of the experiment, while others will not (e.g. new primer pairs will need to be purchased to assess other loci, but adding more populations does not incur more primer synthesis costs).

Cost item	Cost per	Quantity	Total
gDNA extraction of up to 250 flies	$10	8	$80
Primer synthesis	$5	10	$50
Q5 PCR master mix	$4	10	$40
Gel and PCR purification kit	$3	10	$30
Amplicon Sequencing	$75	5	$375
Grand total			$575

Supplementary Note 8: Example of low quality base calls at end of reads

Supplementary Note 8. Per tile sequence quality of two Amplicon-EZ samples. Figures were generated using FastQC v0.11.9 (3). Boxes are colored based on mean Phred base quality score of a given tile-bp position relative to the mean quality score of the entire sample. Dark blue boxes indicate mean Phred scores close to the mean of the sample (Phred scores of 36 to 37). Warmer colors indicate more deviation from the mean Phred score of the sample. Bright red boxes indicate mean Phred scores approximately 10 lower than the mean of the sample (Phred scores of 25 to 28). (a) A representative experimental sample with high quality base calls throughout each sequencing read. Source file name: NF-BIG-1_RESUB_R1_001_fastq.gz. (b) A rare sample with mostly high quality base calls, but containing low quality base calls at the end of some reads. Source file name: NRF-wg-1_R2_001_fastq.gz.

a

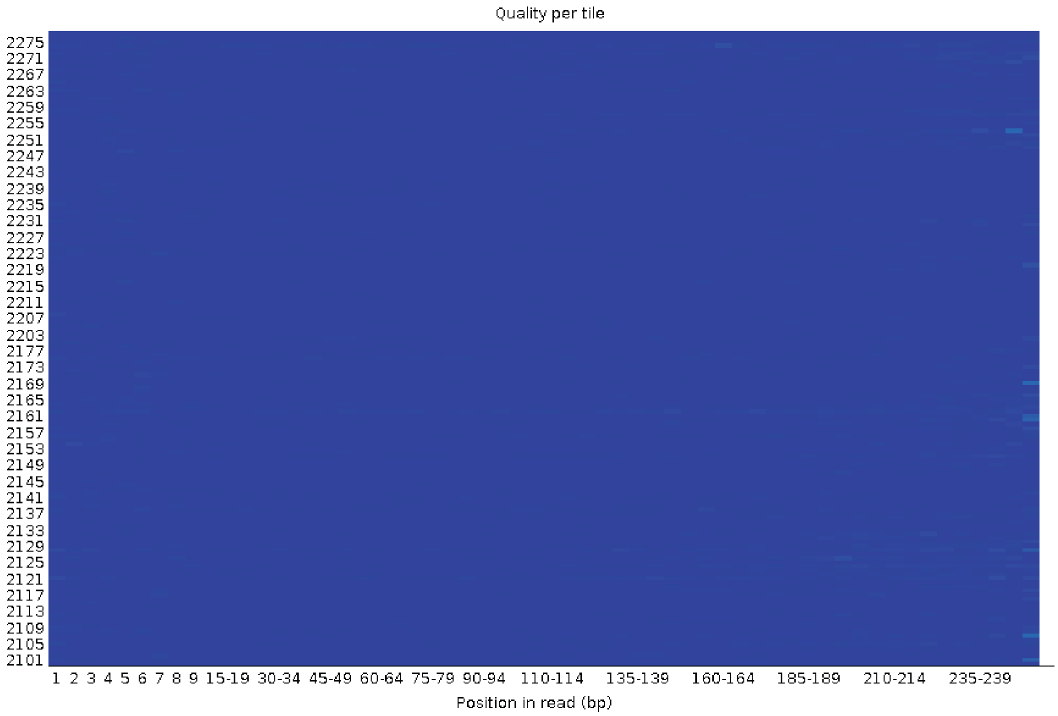

b

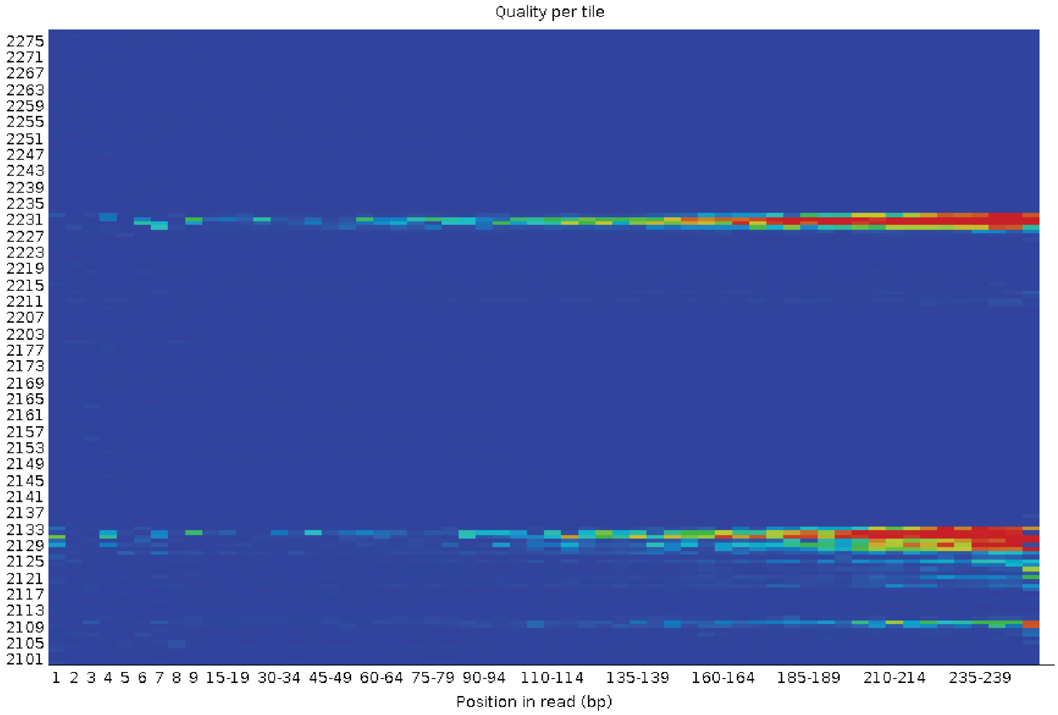

Supplementary Note 9: Genetic variability as a predictor for read merging success rate differences between target genes

Supplementary Note 9. The slope of each of the 10 primer pairs in Supplementary Note 4 are plotted on the y-axis ("read merging success rate"). The three measures of sequence diversity from Supplementary Note 5 are plotted on the x-axes. (a) Average Shannon Entropy per bp vs read merging success rate. (b) Average SNP frequency per bp vs read merging success rate. (c) Percent of bases with SNP frequency >0.01 vs read merging success rate. R^2 values indicate the coefficient of determination for a linear regression between the two variables. All three plots have an R^2 value below 0.3, implying that genetic variability weakly or does not correlate with the read merging success rate.

a

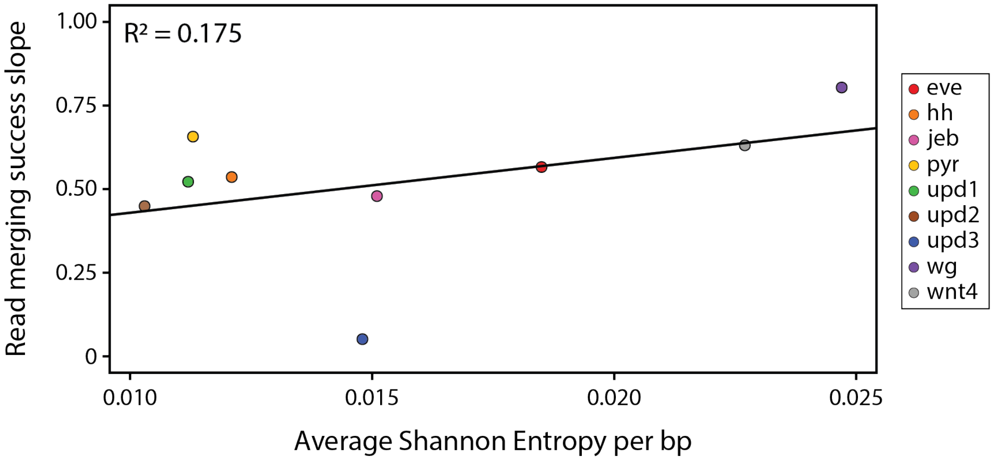

b

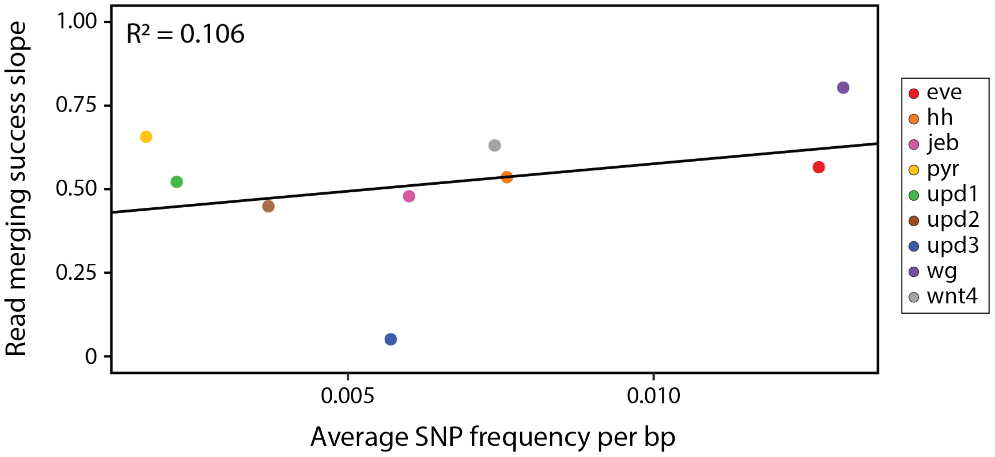

c

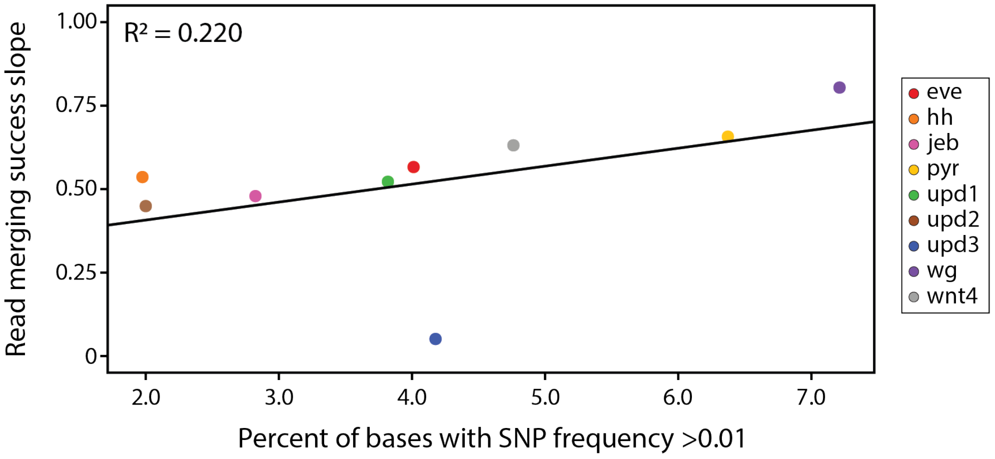

References

1. Zhanshan (Sam) Ma, Lianwei Li, and Ya Ping Zhang. Defining Individual-Level Genetic Diversity and Similarity Profiles. *Scientific Reports*, 10(1):1–12, 2020. ISSN 20452322. doi: 10.1038/s41598-020-62362-8.
2. Joanna C Chiu, Xuanting Jiang, Li Zhao, Christopher A Hamm, Julie M Cridland, Perot Saelao, Kelly A Hamby, Ernest K Lee, Rosanna S Kwok, Guojie Zhang, Frank G Zalom, Vaughn M Walton, and David J Begun. Genome of Drosophila suzukii, the spotted wing drosophila. *G3 (Bethesda, Md.)*, 3(12):2257–71, 2013. ISSN 2160-1836. doi: 10.1534/g3.113.008185.
3. Simon Andrews. FastQC- A Quality Control tool for High Throughput Sequence Data. *Babraham Institute*, 2022.
